# Excessive Attractor Instability Accounts for Semantic Priming in Schizophrenia

**DOI:** 10.1371/journal.pone.0040663

**Published:** 2012-07-23

**Authors:** Itamar Lerner, Shlomo Bentin, Oren Shriki

**Affiliations:** 1 Interdisciplinary Center for Neural Computation, The Hebrew University of Jerusalem, Jerusalem, Israel; 2 Department of Psychology and Interdisciplinary Center for Neural Computation, The Hebrew University of Jerusalem, Jerusalem, Israel; 3 Section on Critical Brain Dynamics, National Institute of Mental Health, Bethesda, Maryland, United States of America; University Of São Paulo, Brazil

## Abstract

One of the most pervasive findings in studies of schizophrenics with thought disorders is their peculiar pattern of semantic priming, which presumably reflects abnormal associative processes in the semantic system of these patients. Semantic priming is manifested by faster and more accurate recognition of a word-target when preceded by a semantically related prime, relative to an unrelated prime condition. Compared to control, semantic priming in schizophrenics is characterized by reduced priming effects at long prime-target Stimulus Onset Asynchrony (SOA) and, sometimes, augmented priming at short SOA. In addition, unlike controls, schizophrenics consistently show indirect (mediated) priming (such as from the prime ‘wedding’ to the target ‘finger’, mediated by ‘ring’). In a previous study, we developed a novel attractor neural network model with synaptic adaptation mechanisms that could account for semantic priming patterns in healthy individuals. Here, we examine the consequences of introducing attractor instability to this network, which is hypothesized to arise from dysfunctional synaptic transmission known to occur in schizophrenia. In two simulated experiments, we demonstrate how such instability speeds up the network’s dynamics and, consequently, produces the full spectrum of priming effects previously reported in patients. The model also explains the inconsistency of augmented priming results at short SOAs using directly related pairs relative to the consistency of indirect priming. Further, we discuss how the same mechanism could account for other symptoms of the disease, such as derailment (‘loose associations’) or the commonly seen difficulty of patients in utilizing context. Finally, we show how the model can statistically implement the overly-broad wave of spreading activation previously presumed to characterize thought-disorders in schizophrenia.

## Introduction

Schizophrenia, one of the most debilitating mental illnesses, is frequently accompanied by thought disorders. Along with other typical symptoms like auditory hallucinations, delusions and general cognitive degradation, patients often exhibit aberrant speech which is presumed to reflect a difficulty in preserving a coherent, meaningful train of thoughts. Among the many sub-types of thought-disorders that have been identified, one of the most common is loosening control on associative thinking, often termed derailment or ‘loose associations’ [1]. During discourse, such patients are often unable to maintain the relevant topic of conversation and repeatedly switch ideas, one after the other, sometimes based on a sporadic word from the previous sentence.

Abnormal associative thinking has often been studied in schizophrenics using the semantic priming paradigm. In a priming experiment, subjects are exposed to two successively presented words, the prime and the target, and are instructed to respond to the target by either naming it aloud or deciding whether it is a real or pseudo word (for a comprehensive review see [2]). The basic finding in such studies is that reaction time is faster and accuracy is higher when the target is preceded by a semantically or associatively related prime compared to an unrelated prime. Many factors have been found to modulate this effect, including the lag between the onsets of the prime and target (termed ‘Stimulus Onset Asynchrony’ or ‘SOA’; e.g., short SOA of around 200 ms or long SOA of 500 ms or more), as well as the exact type of relations between them (e.g., direct relatedness like *table-chair* compared to indirect relatedness like *lion-stripes*, mediated by *tiger*).

**Figure 1:**
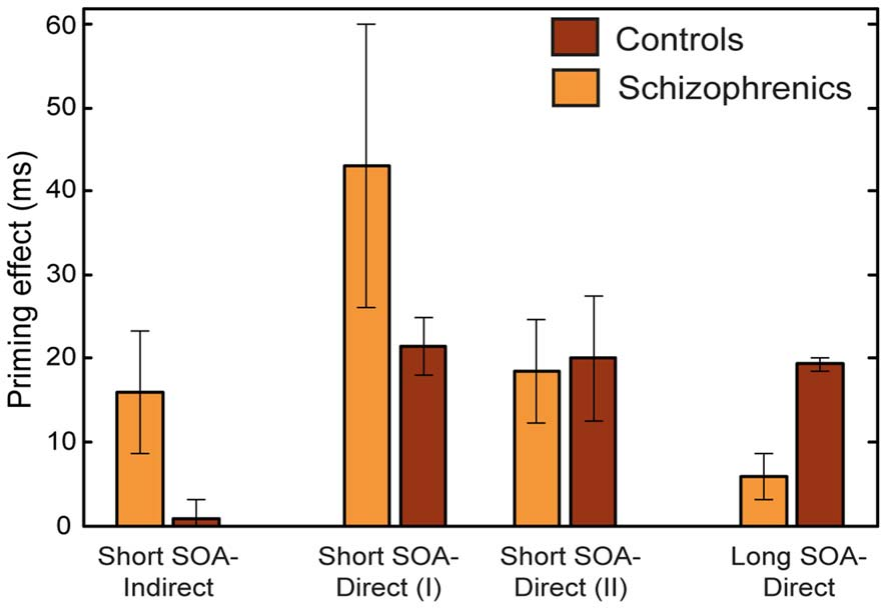
Semantic priming in schizophrenics and controls. Summary of common semantic priming results of controls vs. schizophrenics for short SOA using directly and indirectly related pairs and for long SOA using directly related pairs. Two representative short-SOA results of directly related pairs are displayed to demonstrate the common inconsistency of this condition in the literature. Means and STDs are taken from [8]–[10], [34].

During the past two decades, semantic priming experiments with schizophrenic patients have yielded a wealth of results, roughly summarized as follows (See reviews in [3]–[6]): When the SOA is short and the primes and targets are directly related, some experiments show that schizophrenic patients exhibit augmented priming (hyper-priming) compared with control participants, while others demonstrate equivalent or even reduced priming effects. In addition, at short SOAs schizophrenics usually exhibit augmented indirect priming (using pairs with indirect relatedness) relative to controls, who often do not exhibit indirect priming at all [2]. The hyper-priming effect in schizophrenics is more consistently found using indirectly related pairs than directly related pairs, with the diversity of the direct priming effects hypothesized to stem from differences in the stimuli list, methodology, or from the large variability among patient groups [5]. At long prime-target SOAs schizophrenic patients usually show reduced priming (hypo-priming) relative to controls when directly related primes are used (indirect priming has almost never been examined at long SOAs and therefore cannot be reliably addressed; but see [7] for an exception). All the above effects are more conspicuous for schizophrenics with significant thought disorders compared to schizophrenics whose thought disorders are less severe [3], [7]–[10]. Typical examples of these findings are depicted in Figure 1.

Former models of semantic priming in schizophrenia have addressed only limited aspects of the above findings. Several studies, focusing on hyper-priming, postulated that the enhanced effects in schizophrenics arise from either excessive activation in semantic memory of patients [11] or excessive synaptic pruning leading to reduced connectivity between cortical areas [12]. One study [13] suggested that general associative deficiencies, including certain priming impairments which were not classified as either hyper- or hypo-priming, may result from suboptimal dynamic neuronal thresholds. Another approach was taken by Lavigne and Darmon [14], who showed that altered amounts of dopamine in a cortical network model may lead to both hyper-priming of directly related pairs and hyper- or hypo-priming of indirectly related pairs, depending on the degree of dopamine in the system. However, although informative, these studies did not take under consideration several important modulators of semantic priming in schizophrenia such as SOA and the interaction of SOA with the type of prime-target relatedness; consequently, a major part of the findings reported in the literature is left unaccounted for by contemporary models.

Recently, Rolls and colleagues [15]–[16] have suggested a novel computational perspective on schizophrenia, claiming that many cognitive symptoms of the disease could be explained as a result of impaired attractor dynamics in cortical networks of patients. Specifically, these authors have shown that the well-known hypothesis of NMDA-receptor deficiency in schizophrenia may lead, when implemented in a computational model, to the destabilization of attractor states representing memories and result in uncontrolled switching between these attractors. It was suggested that such excessive instability, when occurring in the temporal lobe where semantic concepts are supposedly stored, may form the basis of thought disorders. Nevertheless, the authors did not link this model to any specific cognitive task known to be impaired in schizophrenics. Following a similar conceptualization to that of Rolls and colleagues [16], in the present study we demonstrate how a recurrent neural network model with excessive attractor instability, possibly stemming from a decrease in NMDA conductance as well as other related biological deficiencies, can, in fact, account for the diversity of schizophrenic priming effects and thus unite many of the previously reported findings within one explanatory mechanism based on a plausible and parsimonious biological impairment.

## Methods

### Model

**Figure 2:**
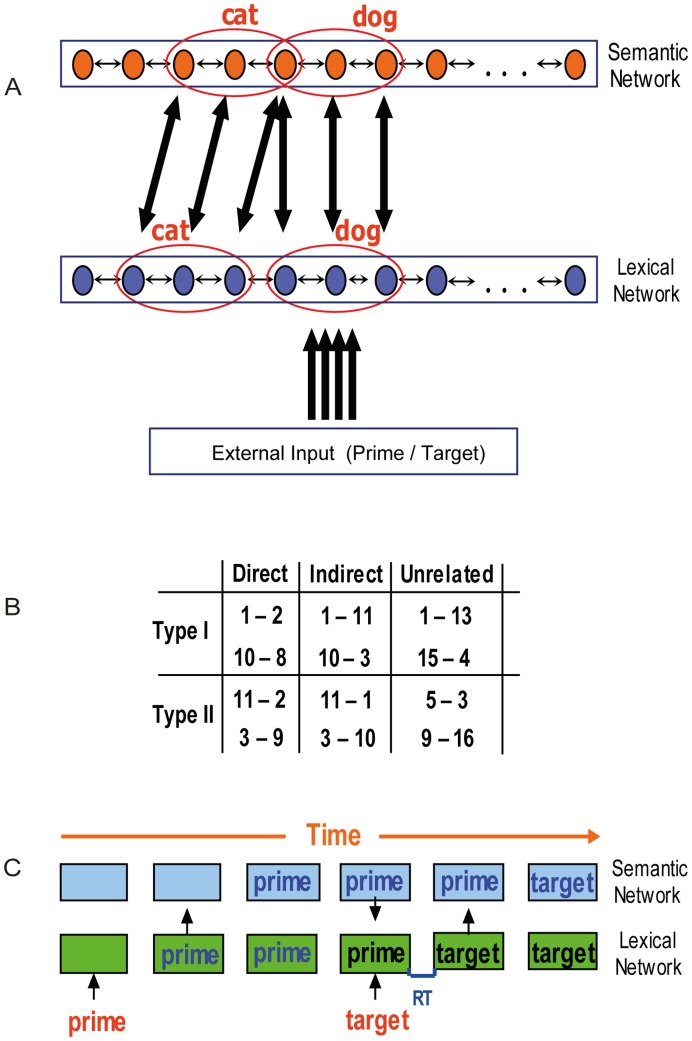
Outline of the model and its dynamics. A: Model Architecture. Patterns representing related concepts are correlated in the semantic network but uncorrelated in the lexical network. Active neurons of two example patterns representing ‘dog’ and ‘cat’ are marked. Connections between networks are from active neurons of a pattern in one network to all the corresponding active neurons in the other network. For simplicity, only some of these connections are drawn. B: Examples of pairs used in the simulation trials, organized by relatedness condition. C: Example of expected chain of events in a semantic priming simulation. Lexical network converges to the prime pattern, followed by convergence of the semantic network. When target appears, the lexical network converges to the appropriate target pattern under the influence of the semantic network. No latching dynamics is assumed in the example.

The currently proposed network is based on the model presented in our previous study [17], [18]. Here, we describe only the main attributes of that network. The model contains two computational layers, one lexical/phonological and the other semantic (Figure 2A). External input representing a visually presented word is fed into the lexical/phonologic layer where the word is recognized. The activity elicited in the lexical layer is fed forward to the semantic layer where the word’s meaning is stored. Importantly, these processes are interactive, so that, in addition to the feed forward transmission from the lexical to the semantic layer, the semantic layer can influence the lexical layer by feedback. The time it takes for the lexical layer to converge on the activity pattern corresponding to the external input is considered as the Reaction Time (RT) for this input, analogue to the recognition time of human subjects.

The lexical and semantic layers are modeled as attractor neural networks with sparse representations and continuous-time dynamics [19]–[21]. Each network is a fully connected recurrent network composed of 500 neurons. Memory patterns encoded to each network are binary vectors of size 500, with ‘1’ indicating a maximally active neuron, and ‘0’ an inactive one. The representations are sparse (i.e., a small number of neurons are active in each pattern). When an external input activates neurons which are part of a specific memory pattern in the network, the activity of the entire network is driven by the internal connectivity to gradually converge to this pattern. The connectivity matrix between the neurons of each layer assures the stability of the patterns. External inputs are always excitatory. The neurons are analog within the range {0,1} and reach binary values when converged on one of the memory patterns. Each neuron obeys a logistic transfer function of its local input *h_i_(t)*:
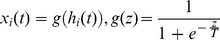
and the dynamics of the local input are given by:



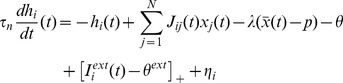



Here, 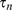 is the time constant of the neuron, *x_j_* is the activity of the *j*-th neuron (with 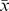 indicating average over all neurons), *J_ij_* is the connectivity weight from neuron *j* to *i*, *N* is the number of neurons (500 in our case), *p* is the sparseness of the representations,
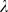
a regulation parameter which maintains stability of mean activation, and 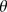 is a constant neuron-activation threshold, which can also be seen as global inhibition [21]. The threshold linear function 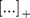 allows the external input to the neuron,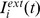, to influence the network activity only if it surpasses some constant external threshold 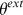. Finally, 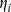 is a noise term drawn from a Gaussian distribution with standard-deviation *η_amp_* and temporal correlations *τ_corr_*. The temporal correlations in the noise were generated by filtering white noise using a low-pass filter, which, for two time points separated by τ ms, took the form:
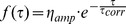



In the semantic layer, memory patterns represent concepts. Relatedness between concepts is implemented as correlations between memory patterns (reflecting the degree of overlap between them). For example, in Figure 2A, the concepts *DOG* and *CAT* are sharing one active neuron, making them slightly correlated. The higher two concepts are related, the stronger their correlation is; unrelated patterns have a correlation near 0.

In addition to the typical stable-state dynamics, the semantic network is also influenced by adaptation mechanisms, which prevent neurons from maintaining a steady firing rate and make the network unable to hold its stability infinitely. As a consequence, with time, the network autonomously leaves the attractor on which it converged and converges to a different one. The process may repeat again and again, with the network ‘jumping’ from one attractor to another. Such type of jumps between attractor states, hypothetically reflecting associative thought chains, was termed ‘Latching Dynamics’ by Treves [22]. It was found that there is a higher probability of network transitions between correlated patterns rather than between uncorrelated ones, since the former require fewer changes in the overall activity [23].

**Figure 3:**
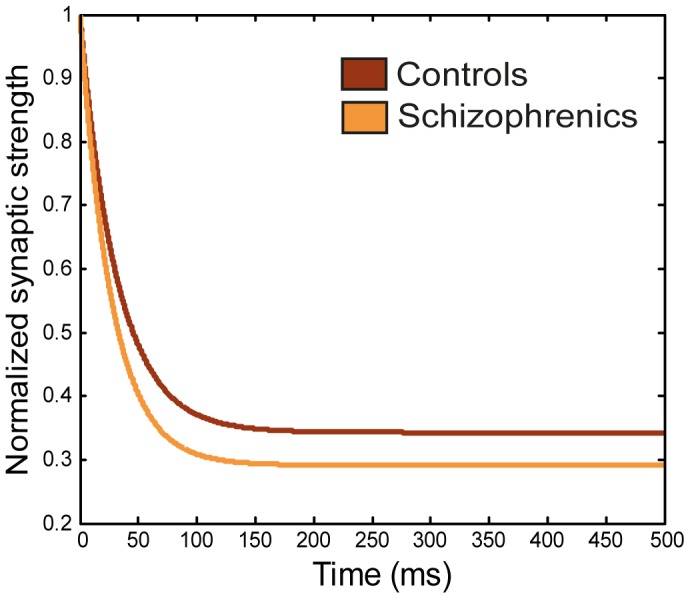
Synaptic depression of schizophrenics and controls in the model. Depression of synaptic strength over time, for the control and schizophrenic network conditions. Results were obtained by simulating the depression dynamics of one synaptic connection when the pre-synaptic neuron was fully activated. The synaptic strength is normalized by the initial (full) strength. Synapses in the schizophrenic network condition show an accelerated rate of depression due to a higher utilization of synaptic resources.

The mechanism by which adaptation is implemented in our network is short-term synaptic depression, which is abundant in many cortical synapses ([24]–[26]; see, however, other implementations of adaptation mechanisms in [13], [23]). In essence, the efficacy of the synaptic transmission between two neurons depends on the history of previous presynaptic activations. For short-term depression, when the presynaptic neuron starts firing a train of spikes, each presynaptic spike is assumed to exploit a certain fraction, *U*, of the available resources (often referred to as the ‘Utilization’ parameter [26]). Consequently, the initially strong post-synaptic reaction becomes weaker and weaker until a plateau level is reached (see brown line in Figure 3). After the transmitter release, the synapse recovers with some typical time constant,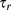, usually in the order of hundreds of milliseconds or even seconds. Specifically in our network, the connectivity from neuron *j* to neuron *i* obeyed the following rule [27]:
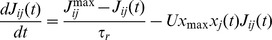
with 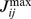
being the common Hopfield connectivity weight for sparse networks [21] and *x_max_* a hypothetical maximum firing rate of a neuron (for example 100 pulses/sec) which adjusts the equation to fit a neural firing rate bounded by 1.

At the cellular level, this depression is often attributed to a decrease in the available presynaptic (e.g., depletion of vesicles) or post-synaptic (e.g., receptor desensitization) resources. Applying synaptic depression in the semantic network causes the efficacy of both the excitatory synaptic connections among the active neurons of an attractor and the inhibitory connections from the active to silent neurons to decrease with time. Consequently, after maintaining stability for a typical interval, the network leaves the attractor and converges to a different one. During this time, the depleted synapses have the opportunity to recover.

In the lexical layer, encoded memory patterns represent words. The dynamics is similar to the one governing the semantic network, with two important differences: There are no correlations between the word patterns in the lexical network (indicating no lexical relations between the words, such as ‘bat’-‘rat’ and ‘cable’-‘table’, mimicking the lack of such relations in typical stimuli of semantic priming experiments) and there are no adaptation mechanisms which cause latching dynamics (resulting in simple steady-state behavior with no associative transitions). The links between the lexical and semantic networks are based on connections between active neurons in corresponding patterns (See Figure 2A). An activated neuron in a certain word pattern in the lexical network sends excitatory connections to all active neurons in the corresponding concept-pattern of the semantic network and vice-versa. Therefore, the activation of one word pattern in the lexical network activates to different extents all related concept patterns in the semantic network, and vice-versa. The bottom-up input to the lexical network, which represents visually presented words, is also excitatory and activates only the neurons that are included in the corresponding word pattern.

Lexical-to-semantic connections are strong but are also subject to synaptic depression with slow recovery time. This allows the lexical network to have a fast, short-enduring influence on the semantic network, allowing it to quickly converge to the appropriate concept pattern and engage in latching dynamics with no further interference (until a new bottom-up external input arrives and the lexical network converges to a new word pattern). Semantic-to-lexical connections are weak and are not suppressed, which allows the semantic network to have a slow and enduring effect on the lexical network. This top-down influence adds up to the bottom-up external influence and allows priming effects to appear: If the meaning of a newly processed word (target) is related to a concept already activated in the semantic network (prime), the lexical network will recognize this word faster than if the target is not related to the prime, because both the bottom-up and the top-down (correlation-dependent) streaming contribute to the recognition process (for a similar conceptualization in an interactive-activation model, see [28]). Lastly, the bottom-up input to the lexical network is constant for as long as a word is visible to the system and is extinguished when the visual word disappears.

### The Schizophrenic Network

Our basic premise in modeling the schizophrenic semantic network comes from the notion that loose associations are caused by an unrestrained associations-chain in semantic memory. Specifically, in our model, this hypothesis took the form of an accelerated rate of transitions in the semantic network, stemming from an inability of the network to maintain steady-state activity to the same duration as in the control condition.

The acceleration of transitions was achieved in our model by increasing the utilization value, *U*, compared to the control condition. When the utilization is large - or, alternatively, when the recovery rate of the synapse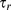 is slow - the attenuation of synaptic resources becomes more pronounced and significantly destabilizes the steady state. Since synaptic resources decay progressively, this attenuation does not typically affect synaptic transmission immediately; rather, it is expressed in longer time scales such that the initial convergence to an attractor is not affected whereas the ability of the network to remain converged is impaired. Both the magnitudes of *U* and 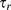 can, therefore, determine the pace of transitions between attractors, with high values of *U* or low values of 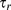 accelerating the latching process (See Figure 3 for a simulated demonstration of the synaptic decay of one connection in the schizophrenic and control semantic networks).

Our premise is supported by previous computational studies suggesting that the maintenance of steady state activity might be seriously flawed in schizophrenics (for a review see [16]). Those studies proposed that NMDA glutamate receptor deficiencies, which are believed to be an important characteristic of the disease [29]–[30], may lead to instability by reducing the firing rate of post-synaptic neurons (due to a decrease in the total synaptic transmission to these neurons) and, consequently, cause the attractor basins of memories to be shallower, rendering them more susceptible to noise. NMDA receptors, which are often modeled as having longer time constants compared to other common glutamate receptors like AMPA (e.g. 100 ms vs. 5 ms, as in [31]), are thought to play an important role in sustaining synaptic transmission rather than in initiating it [32]–[33]. A reduction in NDMA conductance is, therefore, expected to affect synaptic resources at the longer time scales. Since our model does not distinguish between receptor types, the time-dependent reduction in synaptic resources is simplified as an accelerated depression compared to the control case (see Discussion for more details).

### Encoded Patterns in the Simulations

Sixteen different memory patterns, each representing a word/concept, were encoded in the semantic and lexical networks. In addition, a 17^th^ memory pattern was encoded in each network which served as a baseline state to which the networks have been initialized at the beginning of each trial. This baseline ensured that the network would not readily converge to one of the ‘real’ patterns as soon as the trial begins and its stability allowed the network to maintain activity until the prime’s onset. Within each network, all patterns were binary vectors with equal mean activity and a very sparse representation (see Table 1 for specific values).

**Table 1:** Parameters in the model.

Parameter	Semantic Network	Lexical Network
Number of neurons, *N*	500	500
Sparseness, *p*	0.06	0.04
Correlation strength (% of overlapping active neurons out oftotal active neurons in a pattern)	0.066 (Typical) 0.1 (Strong)	0
Neuronal gain, *T*	0.05	0.05
Neuron’s time constant, *τ_n_*	7 [ms]	13 [ms]
Neuronal activation threshold, *θ*	0.02	0.17
Regulation parameter, λ	14.75	27.75
Maximal firing rate, *x*_max_	100 [spks/sec]	100 [spks/sec]
Utilization of synapses within each network, 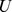 _[within]_	Control: 0.206 [1/spks] Schiz.: 0.2615 [1/spks]	0 [1/spks]
Utilization of synapses between networks, 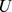 _[between]_	Lexical to Semantic: Control: 0.087 [1/spks] Schiz: 0.1104 [a/Spks]	Semantic to Lexical: Control: 0 [1/spks]Schiz.: 0 [1/spks]
Synaptic recovery time within each network, *τ_r [within]_*	93 [ms]	–
Synaptic recovery time between networks, *τ_r [between]_*	Lexical to Semantic: 1333 [ms]	Semantic to Lexical: –
Input gain between networks (Raw values. Actual valueswere normalized by the number of pre-synaptic activeneurons in a pattern)	Lexical to Semantic: 2	Semantic to Lexical: 0.21
External input gain	0.56	–
Input threshold, *θ_ext_*	1	0.25
Noise amplitude, *η_amp_*	Default: 0.05 Low latching: 0.02	0.025
Noise temporal correlations, *τ_corr_*	17 [ms]	17 [ms]
Convergence threshold	0.95	0.95

**Figure 4:**
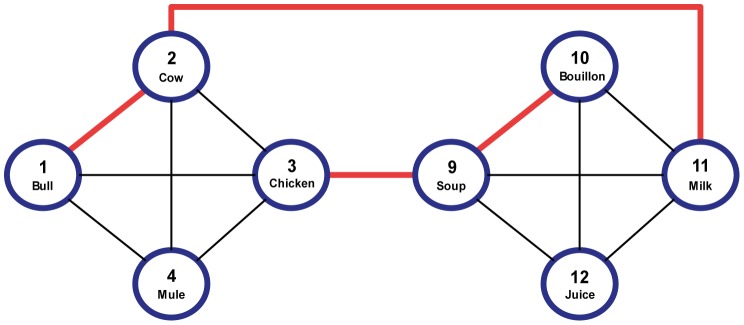
Relatedness between concepts in the simulations. The first and third neighborhoods are shown (The second and fourth neighborhoods had similar connections, with no further connections between the four neighborhoods). Specific words are attached to the concept numbers for easier conceptualization. Connections between concepts are indicated by the lines connecting the circles, with basic-strength relations drawn in black and strong relations drawn in red. The connections form two basic neighborhoods, animals and liquid foods, with some connections crossing between neighborhoods. Word pairs like *Bull*-*Cow* or *Bouillon*-*Soup* are examples of the first-type pairs. *Milk*-*Cow* and *Chicken*-*Soup* are examples of the second-type pairs (see text for details).

In the semantic network, the basic correlations between concept patterns were a priori set as following (Figure 4): Four groups, each containing 4 patterns, formed ‘semantic neighborhoods’ (patterns 1–4,5–6,9–12 and 13–16); each pattern in a neighborhood was positively correlated with the other patterns in the same neighborhood but, with few important exceptions (see below), no correlations existed among the neighborhoods (as all patterns were binary vectors with sparse representations, the overlapping active neurons were actually the major contributors to the correlation. Unrelated concepts were created as patterns with no overlapping active neurons, which actually led to a small negative correlation between them. This small bias from 0 had no significant effects on either the behavior of the network or the results). All correlations within a semantic neighborhood were equally strong with the exception of one pair, which was correlated stronger than the others. For example, in the first semantic neighborhood, patterns 1 and 2 were correlated stronger than patterns 1 and 3, 1 and 4, 2 and 3 and 2 and 4, which were equally correlated. Similarly, patterns 5 and 6, 9 and 10 and 13 and 14 were all strongly correlated.

To produce indirect relatedness between concepts in the semantic network, we slightly altered the above basic structure so that some correlations were introduced between a pattern in one neighborhood and a pattern in a different neighborhood. For example, since patterns 1–4 formed a semantic neighborhood and patterns 9–12 formed a different semantic neighborhood, introducing a correlation between pattern 2 and pattern 11 caused patterns 1 and 11 to become indirectly related (mediated by pattern 2). Similarly, we correlated patterns 3 and 9, 6 and 15, and 7 and 13. The resulting structure led to two important types of concept-patterns in the semantic network (see Figure 4): The first (labeled ‘Type-I’) were patterns that were strongly related to one concept in the neighborhood (e.g. pattern 1, which is strongly related to pattern 2) and were weakly related to the other two (patterns 3 and 4). The other type (labeled ‘Type-II’) were patterns that were strongly related to one concept outside their neighborhood (e.g., pattern 3, related to pattern 9 or pattern 11 related to pattern 2) and weakly related to the other three patterns inside the neighborhood (e.g., patterns 1, 2 and 4 in one neighborhood or patterns 9, 10, and 12, in the other neighborhood).

The above structure was chosen to allow examining whether important variations in priming may result from a specific choice of prime-target pairs. Indeed, previous investigations of semantic priming in schizophrenics have employed very different types of related primes and targets, ranging from pairs chosen for their strong associative relations (e.g., *blaze* - *fire*; [34]) to pairs which mostly share semantic relations such as synonyms and antonyms (e.g., *evil* - *bad*, [7]) or category-exemplars (e.g., *bird*-*robin*; [35]). In our model, associative relations between two concepts are reflected as a high transition probabilty from one concept pattern to another during the latching dynamics process in the semantic network [17]. Since latching is stochastic, transitions from the prime concept can lead the network to converge on a concept matching the upcoming target pattern, but it can also lead the network to jump to other concept patterns. The consequences of these ‘wrong’ transitions are different for type-I and type-II primes: Whereas type-I primes would almost always lead to jumps within the semantic neighborhood, type-II jumps may either lead to jumps within the prime’s neighborhood or to jumps to its strongly related paired word outside the neighborhood. Since the state of the semantic network affects the convergence speed of the lexical network through feedback, this diveristy may lead to variable results in priming experiments when comparing schizophrenics and controls.

In the lexical network, all 17 word patterns were unrelated to each other, representing the lack of phonological/lexical relations between the experimental words (such that only semantic relations would be consequential, as customary in semantic priming experiments). The 17th pattern was, again, the initial state of the network, and was not linked through top-down or bottom up lexical-semantic connections to the baseline pattern in the semantic network (thus forming a ‘neutral’ pattern).

### Experimental Procedure

Simulations of the model were written in MATLAB 10a and were run on an Intel Core 2 Quad CPU Q6600 with 2.4 Ghz and 2 GB of RAM. In all simulations, one numeric step represented 0.66 ms. Details about the parameters of the semantic and lexical networks are presented in Table 1.

#### Simulation 1: short stimulus onset asynchrony

In this simulation we examined whether the acceleration of latching as hypothesized in the schizophrenic semantic network can account for the schizophrenic priming results at short SOA. Using directly related pairs, the experimental results varied across different studies, showing in some hyper-priming, in others hypo-priming and in still others normal priming. In contrast, using indirectly-related pairs the consistent priming pattern is hyper-priming. In attempt to explain the diversity of results in the literature using directly related pairs, we examined whether it might be explained by differences in the type of materials used in different studies. This exploration was based on using different ratios of Type I and Type II related pairs in different experimental conditions.

Each trial consisted of the presentation of a prime followed by a target. Both primes and targets matched existing word-patterns encoded in the lexical network and could either represent a directly related, indirectly related or an unrelated pair (based on the correlation between their corresponding representations in the semantic network). In unrelated trials, primes and targets were chosen from different neighborhoods with no correlations between any of their concepts. In related and indirectly related trials, pairs could either include Type-I or Type-II primes. Only strongly related prime-target pairs were used in the related condition (e.g., patterns 1–2 as prime and target for Type-I and patterns 3–9 for Type-II) and only pairs related strongly through a mediating concept were used in the indirectly related condition (e.g., patterns 1–11 for Type-I and patterns 3–10 for Type-II), mimicking typical stimuli choices in the literature. The ratio of related pairs containing Type-I primes to the total number of related pairs was varied between sessions. We ran five sessions using ratio values ranging from 0 to 1, to grasp potential differences in stimuli lists of previous human experiments. The primes and targets were randomly chosen from within the possible combinations for each relatedness condition (see examples for all experimental conditions in Figure 2B). The simulation was conducted twice, once for the control network and once for the schizophrenic network, thus creating a 3×5×2 design with Relatedness (Related, Indirectly Related, Unrelated), Type-I primes ratio (0, 0.25, 0.5, 0.75, 1) and Condition (Schizophrenic, Control) as independent factors.

Each trial started with the presentation of the prime patterns as an external input to the lexical network for 100 ms. This input was followed by a 100 ms time window in which no input was presented. A second stimulus, serving as the target, was then presented (thus creating a 200 ms SOA; cf. [7], [10]) and persisted until the end of the trial. The RT to a target was measured from its onset and until the convergence of the lexical network on the target attractor. Convergence was defined as the network’s state reaching a 0.95 correlation with the respective memory pattern and a smaller than 0.5 correlation with the other patterns. Figure 2C presents an example of this chain of events in a non-neutral trial (for simplicity, no semantic transitions were assumed in this figure).

The higher adaptation rate of the schizophrenic condition was modeled by an elevated (about 25%) utilization value compared to the control case (see Table 1). Except for this variation, the schizophrenic network was identical to the control one in all aspects and parameters. 300 trials were run for each relatedness condition, mental condition and prime-type ratio, yielding a total of 9000 trials.

#### Simulation 2: long stimulus onset asynchrony

The second simulation was identical to the first one, except that the prime-target SOA was long (950 ms; cf. [8]), and there were no indirectly related pairs. The aim of this simulation was to examine whether the typical hypo-priming of schizophrenics at long SOA conditions using directly related pairs can result from the same accelerated latching performance used in the first simulation. Again, there were 300 trials for each relatedness condition, mental condition and type-I primes ratio, yielding 6000 trials in total.

## Results

### Simulation 1

#### Dynamics of the network

**Figure 5:**
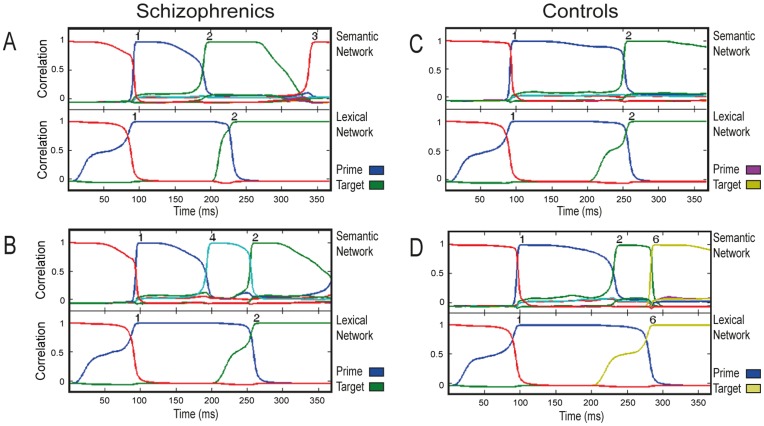
Typical short-SOA trials of controls and schizophrenics in simulation 1. Each graph presents the correlation of the network state, along time, with each of the 17 stored patterns (including baseline; not all lines are seen as they often coincide). Semantic and lexical network are presented separately. A: Typical related trial of the schizophrenic network. The semantic network commits a transition which causes the lexical network to converge quickly to the target state. B: A different related trial of the schizophrenic network, in which the semantic network jumps to a pattern other than the target. C: Typical related trial of the control network. The semantic network maintains stability over the ISI. RT of the lexical network is not as short as in the typical schizophrenic case (shown in A). D: An unrelated trial of the control network, when a transition took place. RT of the lexical network is slower than in the other examples because of the unrelatedness between prime and target.

**Figure 6:**
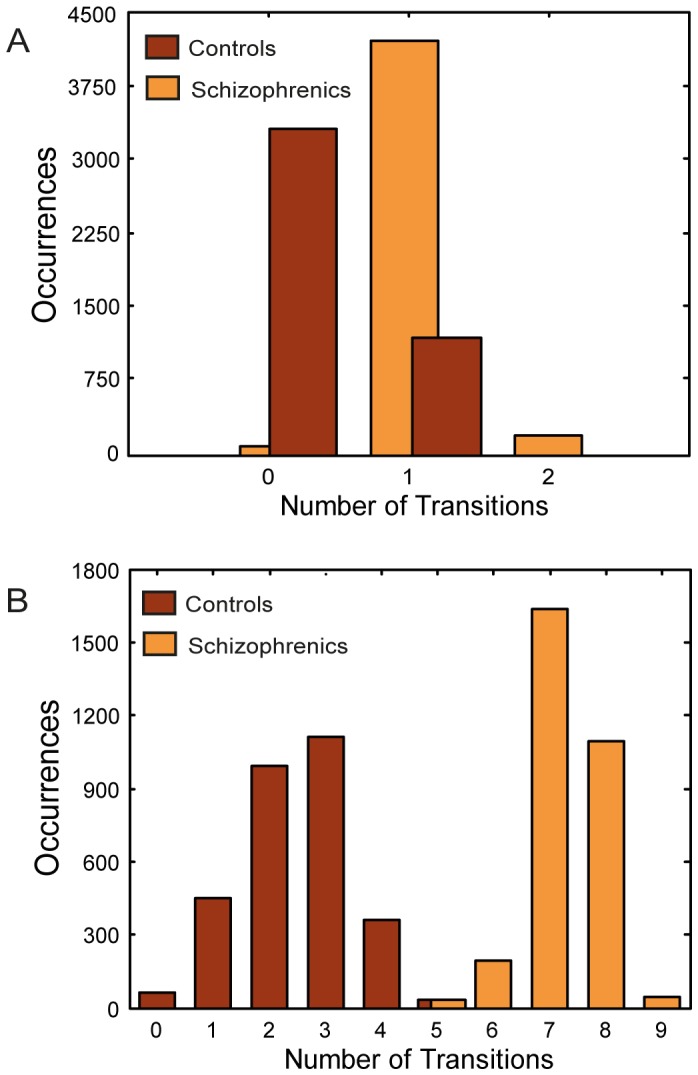
Distribution of transition events for the control and schizophrenic networks. A: Occurrences of transitions at short SOA (Simulation 1). B: Occurrences of transitions at long SOA (Simulation 2).

Figure 5 presents the characteristic time course of activation along a trial in the semantic and lexical networks, for the control and schizophrenic conditions. Correlation of the activation pattern along time for each network with each of its stored patterns (including the real memory patterns and the neutral one) during a trial is presented in different colors, with convergence to a specific pattern indicated by its number appearing on top. As evident in this figure, for both the control and the schizophrenic conditions, the lexical network reacted to the prime by converging to its corresponding memory pattern and remaining stable until the target’s onset. The semantic network in the control condition showed a mixed behavior: Usually, it performed like the lexical network, converging on the appropriate pattern and remaining stable until target onset. In other trials, however, it jumped to another attractor, though these transitions were not common (Figures 5C and 5D). The semantic network in the schizophrenic condition behaved quite differently: After converging to the prime pattern, the network tpyically ‘jumped’ to another attractor before target onset, hence presenting latching dynamics in almost every trial. This jump was often from the prime pattern to its strongly correlated pattern (as in Figure 5A), but other jumps to weakly correlated patterns also occurred (as in Figure 5B). Figure 6A presents histograms of the number of transitions occurring in the control and schizophrenic semantic networks before the convergence of the lexical network to the target-pattern. As evident in that figure, in the majority of the trials, the schizophrenic network committed one transition. In contrast, the control network committed no transitions in about three quarters of the trials, and a single transition in a quarter of them.

#### Priming

**Figure 7:**
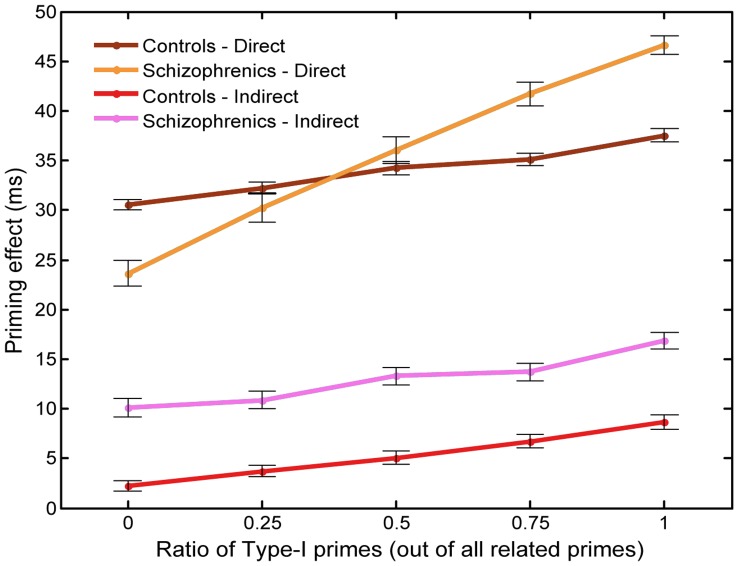
Primings effects in simulation 1. Direct and indirect priming effects for the control and schizophrenic networks as a function of the Type-I primes ratio at short SOA.

The raw RTs were all in the range of 45 to 150 ms. To assess the direct priming effect, we subtracted the mean RTs to directly related targets from the mean RTs to unrelated targets. Indirect priming was computed by subtracting the mean RT to indirectly related targets from the mean RT to unrelated targets. Figure 7 presents the priming effect in the direct and indirect conditions, for the control and schizophrenic networks, as a function of the ratio of Type-I primes. The priming effects were larger for directly related targets compared to indirectly related targets for both networks and for all Type-I primes ratios. Across Type-I primes ratio priming was almost equal between the schizophrenic and control network using directly related pairs (35.65 ms vs 33.93 ms, respectively) but higher for the schizophrenic network than control using indirect semantic relations (12.97 ms vs. 5.27 ms). Most important, however, was the findings that the direct priming effect was modulated differently by the prime-type ratio in the schizophrenic and healthy networks. Specifically, the prime-type ratio caused priming to increase more for the schizophrenic condition compared to the control condition, leading to different results for the two groups. With Type-I prime ratio of 0.5 and above, the direct priming effect was higher for schizophrenics than for control, effectively yielding hyper-priming; with a ratio of 0.25, the direct priming effect was similar for the two groups. When the stimuli list contained only Type-II primes (Type-I ratio  = 0) the schizophrenic network yielded hypo-priming. Thus, the more abundant were Type-I primes among the directly related trials, the stronger was the schizophrenic priming effect compared to controls. Indirect priming, in contrast, increased equally in both groups as a function of Type-I primes ratio and remained stronger in the schizophrenic network regardless of this ratio.

### Simulation 2

#### Dynamics of the network

**Figure 8:**
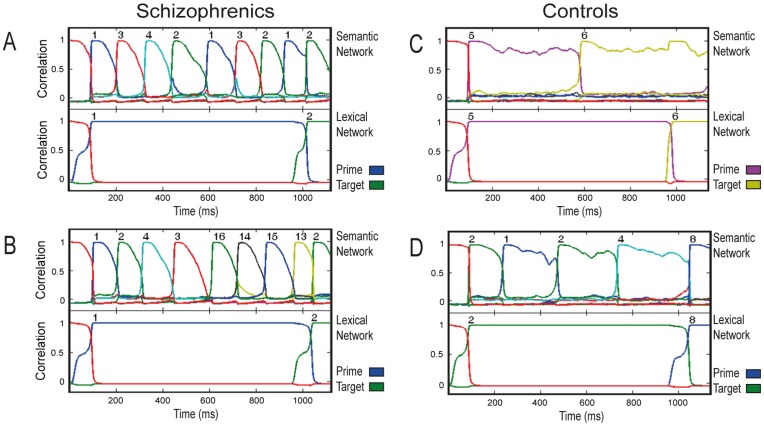
Typical long-SOA trials of controls and schizophrenics in simulation 2. A: Typical related trial of the schizophrenic network. The semantic network engages in a series of semantic transitions within the semantic neighborhood of the prime. B: Another typical related trial of the schizophrenic network, in which the semantic network jumps away from the semantic neighborhood of the prime. The lexical network converges more slowly on the target pattern compared to A. C: Typical related trial of the control network. Transitions are less frequent compared with the schizophrenic network and remain within the neighborhood of the prime during the entire ISI. D: Another typical trial of the control network, with unrelated prime-target pair.

Figure 8 presents the characteristic time course of activation along a trial in the semantic and lexical networks, for the control and schizophrenic conditions. Like in the short SOA case, in both the control and the schizophrenic conditions the lexical network was stable from the moment it converged to the prime and until the onset of the target. In contrast, the performance of the semantic network differed between the two conditions: In the control condition, only a few semantic transitions typically occurred before the target onset, all within the semantic neighborhood of the prime or target (Figures 8C and 8D). In contrast, in the schizophrenic condition, the accelerated latching pushed the semantic network into a series of frequent transitions, which often (though not always) drove the network outside the original neighborhood of the prime or target (Figure 8A and 8B). Figure 6B shows histograms of this number of transitions. Whereas transitions in the control network varied from 0 to 5, with 2 and 3 transitions being the most common cases, the schizophrenic network exhibited many more transitions with 7–8 transitions being most frequent.

#### Priming

**Figure 9:**
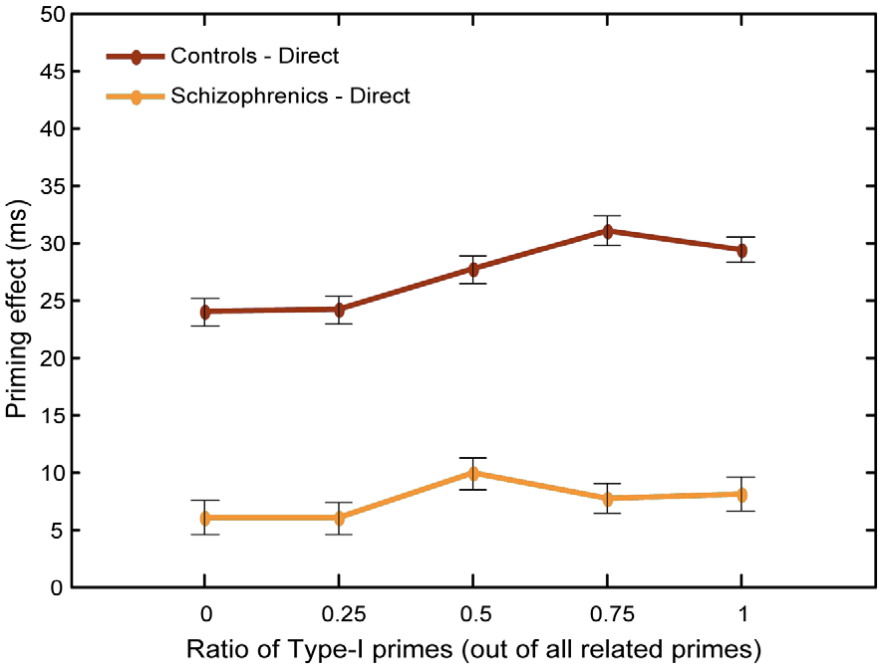
Primings effects in simulation 2. Direct priming effects for the control and schizophrenic networks as a function of the Type-I primes ratio at long SOA.

Figure 9 presents the priming effect of the control and schizophrenic networks as a function of the Type-I primes ratio. For each ratio, the control condition showed consistently larger priming effects compared to the schizophrenic conditition. Across all ratios, the effects were 27 ms and 7.5 ms, respectively.

## Discussion

**Figure 10:**
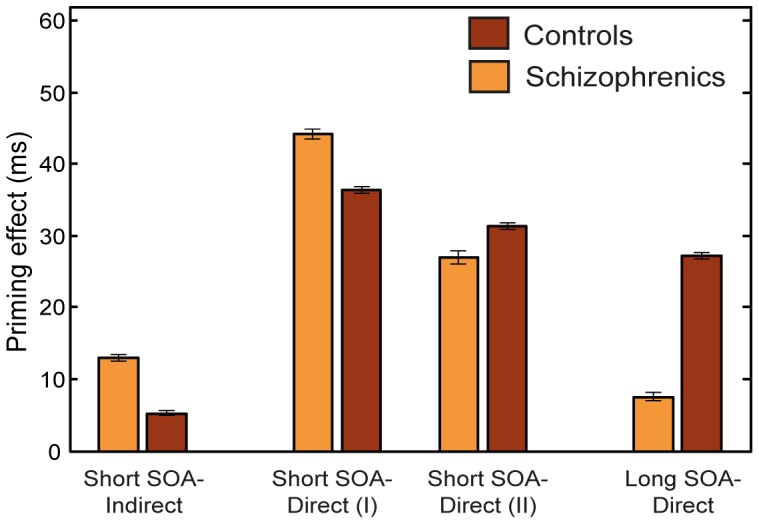
Summary of semantic priming results of simulations 1 and 2. Indirect and long SOA results were averaged across Type-I prime ratio. The two results of direct pairs at short SOA were averaged across either the 0.75 and 2 Type-I prime ratio (I) or the 0 and 0.25 ratio (II), to emphasize their dependency on the stimuli list. Similarly to the experimental results, schizophrenics show hyper-priming with indirectly related pairs at short SOA, diverse results using directly related pairs at short SOA and hypo-priming with directly related pairs at long SOAs.

Replicating our previous results with healthy subjects [17], the current simulations yielded reduced RTs for targets following related primes compared to unrelated primes at both short and long SOAs, thus demonstrating a robust semantic priming effect. However, this priming effect was not equal in the control and schizophrenic conditions; rather, the simulations of the schizophrenic network exhibited the diversity of the priming patterns previously reported in the human-studies literature (for a review see [3]). Specifically, we demonstrated that the size of the semantic priming effect depends on the speed at which transitions from one pattern to another occur in the semantic network, as well as on the statistics of the stimuli used in the experiment. Figure 10 presents a summary of these results mirroring the main findings in the literature (compare with figure 1).

Priming in the current model manifests as a faster convergence of the lexical network on the target, when facilitated by feedback from the semantic network. This facilitation occurs when the semantic network is converged on a pattern correlated to the target, as in related trials, but not when it is converged on an uncorrelated pattern (as in unrelated trials). However, the exact size of the priming effect depends on whether transitions occurred in the semantic network and on the nature of these transitions.

When transitions occur in related trials at short SOA, the first pattern to which the semantic network jumps to is often the upcoming target-pattern (recall that at short SOAs, there is usually time for one transition, at best, before target onset). In this case, all the active neurons in the semantic network contribute to the acceleration of the lexical network convergence when the target appears, and, therefore, facilitation is maximal. When no transitions occur, only a minor set of these neurons (the ones which are shared by the representation of both prime and target) help to accelerate the lexical network’s response. In some trials, the semantic network may jump to a ‘wrong’ pattern – that is, to a pattern other than the target. In such cases, the acceleration of the lexical network could be weakened or even abolished (compared to when no transitions occur), since the correlation of such a pattern to the target might be smaller than the correlation of the prime to the target. The consequence of these ‘wrong’ transitions determines to a large extent whether transitions as a whole will help accelerating RTs or not. Using Type-I primes, in which the primes and targets belong to the same neighborhood (e.g., *bull* - *cow*), ‘wrong’ transitions (e.g., *bull* → *mule*) still remain in this neighborhood and allow priming to occur (even if with a reduced magnitude compared to when no transitions take place). In contrast, Type-II primes belong to a different neighborhood than the targets’ (e.g., *milk* - *cow*; see Figure 4); therefore, using these primes, ‘wrong’ transitions (e.g., *milk* → *juice*) lead the network to converge on concepts which have no relation to the target, thus eliminating priming entirely. Consequently, the more Type-I primes are present among the test stimuli, the more beneficial should transitions be in facilitating the RT to the target. Since in the control condition transitions are not frequent at short SOAs whereas in the schizophrenic condition they are common, the consequence of transitions is higher in the schizophrenic network. As a result, larger priming effects of directly related primes are found in the schizophrenic condition compared to control when Type-I pairs are abunduant in the stimuli list whereas the opposite outcome is evident when Type-II primes are common.

In contrast to direct priming, indirect priming is entirely dependent on the existence of semantic transitions. Since there is no correlation between targets and indirectly related primes, the convergence of the lexical network on the target cannot be facilitated if the semantic network maintains stability on the prime’s pattern. If, however, a transition occurs, then the semantic network may jump to a pattern that is correlated with the upcoming target, and therefore facilitate its recognition (e.g., *bull* → *cow*, when the target is *milk*). ‘Wrong’ transitions (e.g., *bull* → *mule*) cannot facilitate recognition and thus they maintain the null effect of the original prime. In other words, when the prime-target relation is indirect, a transition can, occasionally, facilitate target recognition relative to the case when no transitions occur, but never delay it. Accordingly, regardless of whether type-I or type-II primes have been used in the simulation, averaging over all trials, the schizophrenic network always showed hyper-priming of indirectly-related pairs compared to the control network, which replicates similar findings in the literature. This finding goes along with the experimental result of a higher consistency of indirect over direct hyper-priming in schizophrenic patients.

Contrary to the short SOA results, when the SOA was long the priming effect of the schizophrenic network was consistently smaller compared to the control network (and was also smaller compared to priming effect of the schizophrenic network in Simulation 1). The long SOA allowed the schizophrenic semantic network to engage in many transitions compared to the control network. When many transitions occur, it is highly probable that at least one of them would lead the network to converge on a concept belonging to a neighborhood different than that of the prime or target. Since the time taken by the lexical network to converge on the target is facilitated only if the semantic network is converged on a concept related to this target, the switch to an unrelated neighborhood eliminated the priming effect. Indeed, in some of the trials, the transitions were restricted to the original neighborhood of the prime even in the schizophrenic network; therefore, the priming effect of the schizophrenic condition was not eliminated completely. On average however, the priming effect was reduced relative to control where fewer transitions have occured and the probability of remaining inside the original neighborhood was high. Note also that since this mechanism is common to all prime types, the type-I primes ratio did not modulate this effect.

### Relation to Previous Models of Semantic Memory in Schizophrenia

**Figure 11:**
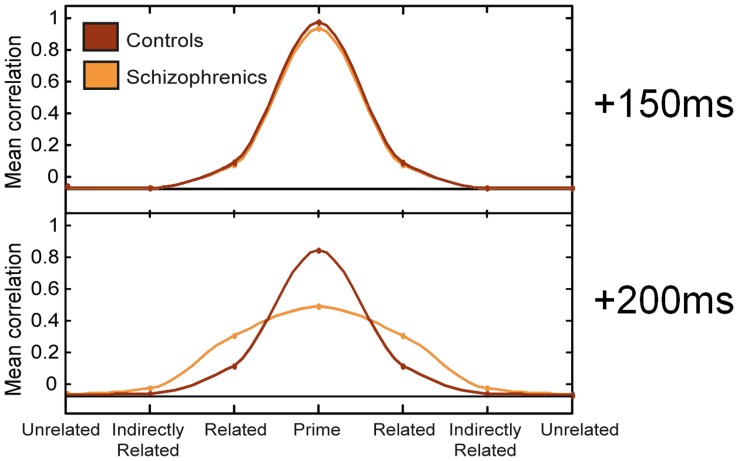
Statistical spreading activation of the control vs. schizophrenic networks. The mean correlation across trials of the network state with the prime concept (1), its strongly related concept (2), indirectly related concept (9) and an unrelated concept (16) was computed for 150 ms and 200 ms after prime onset, separately for the control and schizophrenic network. For presentation purposes, the values of the related, indirectly related and unrelated patterns were replicated twice and interpolation was performed between each value in order to get a smooth symmetric wave-like graph. Data is based on the high Type-I prime ratio data from simulation 1. Only trials when the prime was pattern 1 were considered. See [17] for details.

The acceleration of semantic transitions in our model can be linked to a former (qualitative) theory of aberrant automatic activation in semantic memory of schizophrenics [11]. This theory is based on the well-known spreading activation model [36], which assumes that when a concept in semantic memory is activated, its activation automatically spreads to semantically and associatively related concepts as a wave spreading on water, thus giving them a ‘head-start’ over unrelated concepts when accessed as targets in a priming experiment. Based on this theory, Spitzer [11] suggested that schizophrenic semantic memory is characterized by a faster and further-spreading wave stemming from the prime-concept, which augments the activation of both directly and indirectly related concepts, hence leading to hyper-priming. In our previous study [17] we have demonstrated that the mean correlation over trials between the state of the semantic network and its various memory patterns can be seen as statistically realizing a spreading activation wave. Using these terms, accelerated transitions can be seen as precipitating the spreading of activation. As a result, at any given time, the schizophrenic activation wave should be more spread (that is, reach more distant points in the concept space) than its corresponding normal wave. In this way, our model realizes Spitzer’s original speculation. Figure 11 presents the control and the schizophrenic ‘wave’ at two different time points after prime onset (the data was taken from the highest type-I primes ratio condition of the first simulation). As can be seen, in line with Spitzer’s suggestion, the schizophrenic semantic activation is further-spread than that of the normal semantic activation.

An additional interpretation of the instability of the schizophrenic network addresses the use of strategies which are hypothesized to influence semantic priming (for a review see [2]). As we previously discussed, these strategies may be linked to the subjects’ control over their semantic transitions [18]. Such control was attributed to the ability of subjects to change the values of several network parameters that influence transition tendencies such as the degree of noise in the system (representing the degree of focused attention by the subject). However, when transitions in the network are accelerated due to enhanced utilization of synaptic resources (as in the schizophrenic semantic network), the control over transitions is insufficient: The control parameters are unable to regulate the network’s jumps if the network itself is unstable. Therefore, although not directly tested in the present study, based on this interpretation the peculiar semantic priming effects in schizophrenia might also reflect deficiency in utilizing controlled processes operating in this task (cf. [37]).

### Relation to other Schizophrenic Symptoms

The acceleration in semantic transitions in our network can be linked to other well-documented symptoms in schizophrenia. First, and most obvious, are the notorious ‘loose associations’. In terms of our model, loose associations in speech stem from a failure to preserve stability of attractor states, representing concepts, during discourse. As a result, the semantic network rapidly jumps from one concept to another and quickly looses the initiating topic of the conversation, much like the way the priming effect diminished at long SOAs in our simulations. The same mechanism holds for ‘flight of ideas’, which is usually defined as a severe episode of loose associations. It might also explain the ‘pressured speech’ that characterizes some schizophrenic patients (defined as an increase in the amount of spontaneous speech) if we assume that in order to compensate for their inability to hold concepts for long, these patients attempt to equate their speech rate to their accelerated association rate. Our model can further be related to the finding that schizophrenics suffer from problems in utilizing context (e.g., [38]–[39]). Such results are usually demonstrated in tasks in which a correct reaction to a target stimulus depends upon the context in which the target was presented (frequently established by a cue). Reduced ability of schizophrenics to utilize context (the cue) to their benefit has been frequently reported. For example, in a version of the Continuous Performance Task, the AX-CPT [39], subjects observe different letters in succession and need to respond when they see the letter ‘X’, but only when it follows the cue letter ‘A’. Schizophrenics show a particular context deficit in this task, indicated by responding to the target even when it is not preceded by the correct cue (that is, their results show a smaller difference between hits and false alarms compared to control performance). In terms of our model, the reason for this reduced ability to integrate the target and the context is that the representation of the cue ‘A’ in the schizophrenic network is lost due to the network’s instability. As a result, when patients encounter the target ‘X’, they often may not remember whether it followed the cue ‘A’ or not and be forced to rely on guessing, thus frequently responding to ‘X’ regardless of context. In other words, our model suggests that schizophrenics’ difficulty to utilize context stems from the same mechanism which causes their decreased priming effect at long SOAs. Lastly, schizophrenics are known to have working memory deficits [40]–[41] which can be demonstrated in tasks such as the n-back [42]–[43], digit span [44] and the Brown-Peterson task [45]. If memory is defined as the ability to preserve representations for a sufficient period of time, then, once again, instability of attractors as the one suggested in the current work is compatible with such deficits.

### Caveats and Future Directions

Our findings depend on one core hypothesis, namely, that the schizophrenic semantic system is unable to hold concepts for a long period of time and quickly jumps from one concept to another. We implemented this instability as an increased rate of synaptic utilization, hypothetically stemming from the reported deficiency in NMDA receptors in schizophrenia [29]. The rate of synaptic utilization is usually considered to reflect either pre-synaptic mechanisms, like availability of synaptic vesicles, or post-synaptic mechanisms, such as desensitization of receptors [27]. Naively, our hypothesis could be viewed as suggesting a post-synaptic factor (namely, NMDA deficiency) as the sole contributor to the accelerated depression rate. However, recent in-vivo studies have shown that blockade of post-synaptic receptors may lead to an increased secretion of neurotransmitter from presynaptic terminals, reflecting a compensatory mechanism attempting to maintain homeostatic plasticity [46]. Therefore, the postsynaptic NMDA deficiency could actually result in increased presynaptic activity leading to exhaustion of presynaptic resources. Moreover, depletion of glutamatergic stores leading to enhanced synaptic depression has also been directly suggested in relation to schizophrenia: Several studies have shown that specific proteins known to be impaired in schizophrenics, such as the phosphoprotein Synapsin II, are involved in glutamatergic synaptic plasticity and that disabling these proteins in knockout mice leads to enhanced synaptic depression due to a decrease in the vesiclar reserve pool in these synapses [47]–[48]. Hence, the increase in synaptic utilization hypothesized in our model can be accounted for by both pre- and post-synaptic mechanisms.

It is important to stress that while our model reasonably replicates the priming patterns reported in human experiments with both typical controls and schizophrenics, it does so by using a very simplified network structure which does not address all aspects of word-processing. Importantly, the model does not replicate either the magnitude of the absolute RTs typical to the task (which are approximately 500–600 ms for control subjects) or the common finding of general slowed responses of schizophrenics compared to controls (e.g., [49]–[50]). Recall, however, that in addition to word-recognition time, human RTs reflect the time necessary for choosing and executing the response, including the duration of the actual motor movement and, in lexical decision tasks, also the time it takes to make a decision regarding the lexicality of the target. Considering the well-established finding of slowed motor responses of schizophrenics compared to controls, it is reasonable to assume that the difference in total RTs between the groups can be attributed mainly to those later stages of processing. Since our model does not include such stages, it is obvious that absolute RT differences between the control and schizophrenic conditions cannot be evident in our results. In this regard, some authors suggest that hyper-priming in schizophrenics might be an artifact of the general slowness characterizing those patients (e.g., [4]); in other words, that the slow RTs interact with target recognition rather than being additive to it, as we suggest. While this issue is still debated in the literature, there is some evidence against it. First, hyper-priming was evident also when using relative-priming measures (in which the priming effect is scaled by the mean unrelated RTs) instead of simple difference scores [7]. If hyper-priming was an artifact of the lengthened RTs, this procedure should have eliminated the effect. Second, hyper-priming was also found comparing error rates, which obviously cannot be accounted for by general slowness [51]. Third, hyper-priming was robust even when the RTs of schizophrenics were compared to their predicted values using regression analysis based on the RTs of a control group (e.g., [9]). Fourth, if hyper-priming was an artifact, it should not have been more conspicuous using indirect priming measures compared to direct measures, contrary to the pattern of findings in the literature. Lastly, had RT slowness interacted with target recognition, typical results such as hypo-priming at long SOAs would have been less likely obtained (see also [52]).

An obvious limitation of the model presented in the current study is its size. In contrast to the present implementation, semantic memory of humans contains thousands of neighborhoods, each including hundreds of concepts. The exact nature of latching dynamics is known to depend on various characteristics of the network, including the total number of stored patterns, the correlations between the patterns and the ratio between the number of stored patterns and the number of neurons, known as the network’s capacity [53]–[54]. These parameters influence the degree to which transitions follow the correlation structure of the network and even whether transitions occur at all. Therefore, when implementing our model in a larger (hence, more realistic) network which includes many more patterns and neighborhoods, it would be imperative to correctly scale up the model’s parameters in order to maintain the same dynamics as in the present study and avoid unwarranted phase transitions to different dynamical regimes.

Finally, we note that the results of our simulations carry a prediction which can be tested in future human studies. If, indeed, the contradicting results of direct priming in schizophrenics stem from pair-type differences in the stimuli, it would be possible to perform a semantic priming experiment in which this factor is manipulated to produce different levels of pair-type ratio. In one condition, most pairs in the items-list would consist of words which belong to the same semantic neighborhood and share many other related concepts (like type-I primes in our simulation). In the other condition, pairs would be constructed from strongly related words which are not part of the same semantic neighborhood and do not share additional semantically or associatively related words (like type-II primes). The prediction, based on the present results, is that in the first condition schizophrenics will exhibit hyper-priming with directly related pairs, while in the second condition schizophrenics will exhibit normal or hypo-priming. In addition, the model predicts that indirectly related pairs would yield hyper-priming in both conditions. Another prediction follows our assumption that a common mechanism is responsible for both hypo- and hyper-priming. If true, then we should find a reverse correlation between the priming effects which schizophrenics exhibit in short SOA conditions and their respective priming effect at long SOAs (provided, of course, that the short SOA yields hyper-priming). An appropriate experiment using both SOAs can thus be planned to validate this hypothesis.

In conclusion, semantic priming in schizophrenia has yielded different and sometimes contradicting results over the past twenty years. We have shown how this spectrum of findings can be accommodated by a single biologically-plausible mechanism relating attractor instability to the semantic network of patients, and discussed how this mechanism can be connected to other cognitive symptoms of the disease. Attractor instability leading to abnormal network transitions may also be linked to additional well-known schizophrenic thought-disorders (see [1]). For example, a change in the type of transitions, rather than in their rate, may be related to symptoms like clanging (when sounds, rather than meaning, determine how words are combined into a sentence), perseveration (where the same word or sentence is repeated again and again), or semantic paraphasia (where one word in a sentence is replaced by another, inappropriate word). Future experimental and computational work could examine these hypotheses, as well as several of the predictions arising from the current model.
